# Interactive Dual Attention Network for Text Sentiment Classification

**DOI:** 10.1155/2020/8858717

**Published:** 2020-11-03

**Authors:** Yinglin Zhu, Wenbin Zheng, Hong Tang

**Affiliations:** ^1^College of Software Engineering, Chengdu University of Information Technology, Chengdu 610225, China; ^2^Software Automatic Generation and Intelligent Service Key Laboratory of Sichuan Province, Chengdu 610225, China; ^3^College of Engineering, Sichuan Normal University, Chengdu 610068, China

## Abstract

Text sentiment classification is an essential research field of natural language processing. Recently, numerous deep learning-based methods for sentiment classification have been proposed and achieved better performances compared with conventional machine learning methods. However, most of the proposed methods ignore the interactive relationship between contextual semantics and sentimental tendency while modeling their text representation. In this paper, we propose a novel Interactive Dual Attention Network (IDAN) model that aims to interactively learn the representation between contextual semantics and sentimental tendency information. Firstly, we design an algorithm that utilizes linguistic resources to obtain sentimental tendency information from text and then extract word embeddings from the BERT (Bidirectional Encoder Representations from Transformers) pretraining model as the embedding layer of IDAN. Next, we use two Bidirectional LSTM (BiLSTM) networks to learn the long-range dependencies of contextual semantics and sentimental tendency information, respectively. Finally, two types of attention mechanisms are implemented in IDAN. One is multihead attention, which is the next layer of BiLSTM and is used to learn the interactive relationship between contextual semantics and sentimental tendency information. The other is global attention that aims to make the model focus on the important parts of the sequence and generate the final representation for classification. These two attention mechanisms enable IDAN to interactively learn the relationship between semantics and sentimental tendency information and improve the classification performance. A large number of experiments on four benchmark datasets show that our IDAN model is superior to competitive methods. Moreover, both the result analysis and the attention weight visualization further demonstrate the effectiveness of our proposed method.

## 1. Introduction

Sentiment analysis has been a hot topic in the field of Natural Language Processing (NLP) in recent years. With the rapid development of social networks and e-commerce, a large amount of text data with user sentiments has been generated on the Internet. Sentiment analysis for these data has significant application value [1–3]. Text sentiment classification is a subtask of sentiment analysis which aims to identify the sentiment polarity (e.g., positive and negative) of a text [4].

Traditional machine-learning-based sentiment classification methods mainly focus on artificially designing a set of features, such as sentiment lexicon or bag-of-words features, to train classifiers [5]. However, this type of method is usually time-consuming and laborious.

In contrast, deep learning methods can learn the feature representation automatically instead of hand-crafted features, which have been used in various NLP tasks such as machine translation [6], reading comprehension [7], and sentiment classification [8–10]. Word2Vec [11] and GloVe [12] are word embedding techniques that are often used in deep neural networks for word feature representation. However, the Word2Vec and GloVe methods give static and context-independent word vectors, which cannot well represent the semantics of words in different contexts. Recently, the Bidirectional Encoder Representations from Transformers (BERT) language model [13, 14] was proposed, which can generate context-aware dynamic word embedding representation and can model context semantics better [15].

Although context-aware semantic representation can be obtained through the BERT language model, the expression of sentimental tendency is still insufficient. Some studies have integrated linguistic resources (e.g., sentiment lexicon) into models to improve the sentimental tendency expression ability of neural networks [16–18]. Nevertheless, these studies have not adequately considered the possible interaction between contextual semantics and sentimental tendency.

This paper proposes a novel model called Interactive Dual Attention Network (IDAN), which is intended to utilize the interaction between contextual semantics and sentimental tendency information for sentiment classification.

First, we design an algorithm combining sentiment lexicon, intensity, and negative words to extract sentimental tendency information from text. The context-aware dynamic word embedding representation obtained through the BERT pretraining model is used as the embedding layer of IDAN. Next, we use two Bidirectional LSTM [19] (BiLSTM) networks to learn the long-range dependencies on contextual semantics and sentimental tendency information, respectively. Since the attention mechanism allows the network to focus on the important parts of the text sequence [20, 21], two types of attention mechanisms are implemented in IDAN. One is multihead attention [22], which is the next layer of BiLSTM and is used to learn the interactive relationship between contextual semantics and sentimental tendency information. The other is global attention [21] that aims to make the model focus on the important parts of the sequence and generates the final representation for classifier.

The main contributions of this paper are as follows:

- An architecture of Interactive Dual Attention Network (IDAN) is proposed, which aims to implement interactive learning between contextual semantics and sentimental tendency information for sentiment classification
- An algorithm to extract sentimental tendency information is proposed
- IDAN is extensively evaluated on four benchmark datasets. Experimental results demonstrate that IDAN outperforms the competitive methods

The rest of this paper is organized as follows. In Section 2, related work of sentiment classification is introduced. Section 3 presents the details about the IDAN architecture and its implementation. Section 4 gives the experimental result and analysis. Finally, we conclude our research in Section 5.

## 2. Related Work

In this section, we will briefly introduce traditional methods for sentiment classification and focus on reviewing deep learning methods.

### 2.1. Traditional Methods

Traditional lexicon-based methods use existing resources such as sentiment lexicons and some linguistic rules to identify the sentiment polarity of text [23, 24]. However, these methods rely heavily on the construction of sentiment lexicons; thus there are few methods that only use lexicons for sentiment classification.

The key of sentiment classification methods based on traditional machine learning is to manually design suitable features for classifiers. Pang et al. [5] first proposed a standard machine learning method to solve sentiment classification problems, in which they attempted to construct different features for three classifiers: Naive Bayes (NB), Maximum Entropy (ME), and Support Vector Machine (SVM). Their experimental results show that SVM combining with unigram features is better than NB and ME algorithms. Furthermore, lexicon information was integrated with SVM to improve the performance of sentiment classification [25].

### 2.2. Deep Learning Methods

Due to the powerful expression ability, deep learning models have achieved remarkable results in numerous fields. For NLP, Recurrent Neural Network (RNN) [19] is quite popular because it can handle variable-length sequences well. Thus, RNN is usually used as the basic network structure of sentiment classification [26]. On the other hand, CNN has achieved excellent results in the field of computer vision [27]. In addition, Kim [28] also used CNN for sentiment classification, which shows that unsupervised pretraining of word vectors may be an important ingredient for NLP.

Furthermore, Wang et al. [29] proposed an architecture that combines CNN and RNN for sentiment classification. This architecture makes use of the local features captured by CNN and the characteristics of long-distance dependencies learned through LSTM or Gated Recurrent Unit (GRU). Tang et al. [30] proposed a model that encodes the intrinsic relations of sentences in semantic meaning, which uses LSTM or CNN to obtain sentence representations and then uses gated recurrent neural networks to aggregate them to obtain document representations. Recently, attention mechanism has been successfully applied in sentiment classification tasks. Yan and Guo [31] proposed a method for text classification using contextual sentences and attention mechanism. Yang et al. [32] proposed a Hierarchical Attention Network (HAN) for document sentiment classification, in which the model can selectively focus on important single words or sentences when constructing the document representation.

In order to enhance sentimental tendency expression, some studies have integrated linguistic resources or some external knowledge into models to enable the network to learn sentiment-specific expressions. Tang et al. [33] encoded sentiment information into the continuous representation of words to learn Sentiment-Specific Word Embeddings (SSWE), which is more suitable for sentiment classification tasks. Qian et al. [16] proposed linguistically regularized LSTM for sentence-level sentiment classification, in which the proposed model addressed the sentimental shifting issue of the sentiment, negation, and intensity words. Besides, some studies also incorporated external knowledge (e.g., sentiment lexicons) into deep learning models for sentiment classification [17, 18, 34].

More recently, Lei et al. [35] proposed a hierarchical sequence classification model based on BERT and applied it to microblog sentiment classification. However, these methods have not considered the possible interaction between contextual semantics and sentimental tendency.

Therefore, our proposed IDAN method uses the context-aware word embedding as the embedding layer and combines it with BiLSTM as well as attention mechanisms, which aims to conduct semantic modeling for a specific context and learn the interactive representation between contextual semantics and sentimental tendency information.

## 3. The Proposed Approach

In this section, we will first introduce the overall architecture of our IDAN briefly and then describe the details of the proposed method.

**Figure 1:**
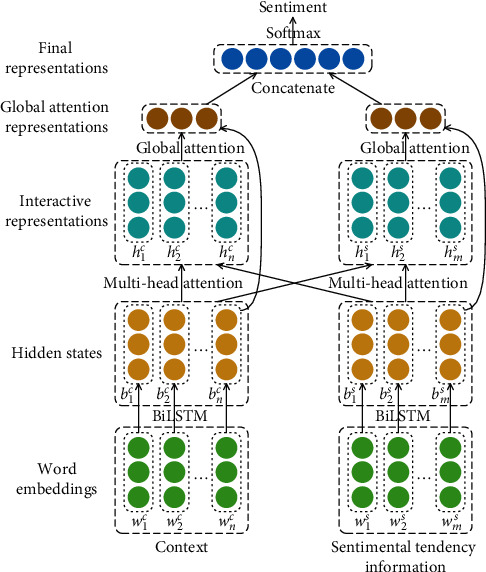
The architecture of IDAN.

The overall architecture of the IDAN model is shown in Figure 1. The model contains two input parts: context and sentimental tendency information, which model contextual semantics and sentimental tendency, respectively. The hierarchical structure of the model is divided into five layers. The first one is the embedding layer, which converts the text sequence into a word embedding matrix. Then there is the BiLSTM layer that is used to model the semantic representation in long sequences. The third layer is the interaction layer, which is used to learn the interactive representation of contextual semantics and sentimental tendency information. The fourth layer is the global attention layer, which aims to combine the last output of BiLSTM to capture important information of sentimental polarity in the sequence after interactive learning. The last layer is the output layer with a soft max classifier.

### 3.1. Sentimental Tendency Information Extraction

The text sentimental tendency information elements are the combination of words or phrases with sentimental tendency. In order to extract these elements, some external resources such as sentiment, intensity, and negative lexicon are utilized.

Here, we denote the set of sentiment, intensity, and negative lexicon by *S*, *I*, and *N*, respectively. Consider a dataset *C* containing *K* texts, in which *c*_*i*_ represents the *i*-th text. We scan the text in order and define a continuous word sequence according to the *j*-th word *w*_*j*_ as *s*(*w*_*j*_)=*w*_*j*−2_*w*_*j*−1_*w*_*j*_. The corresponding sentimental tendency element *e*_*j*_ can be obtained by the following extraction criteria:

(1) $$\begin{array}{c} e_{j}\leftarrow \left\{\begin{array}{cc} s\left(w_{j}\right), & s\left(w_{j}\right)\in N⊗I⊗S,\\ s\left(w_{j}\right), & s\left(w_{j}\right)\in I⊗N⊗S,\\ w_{j-1}w_{j}, & s\left(w_{j}\right)\in \bar{N}⊗I⊗S,\\ w_{j-1}w_{j}, & s\left(w_{j}\right)\in \bar{I}⊗N⊗S,\\ w_{j}, & w_{j-1}\notin I\cup N,w_{j}\in S,\\ \emptyset , & w_{j}\notin S, \end{array}\right. \end{array}$$

where ⊗ means the Cartesian product of two sets and $\bar{N}$ and $\bar{I}$ denote the complements of sets *N* and *I*, respectively. The pseudocode of extracting procedure is given in Algorithm 1.

**Remark 1 .**

Since *N*∩*I*=∅, *N*∩*S*=∅, and *I*∩*S*=∅, there is no conflict according to the extracting criterion.

**Remark 2 .**

We think that the sentiment word is the most important tendency information; thus each element *e* must contain a word coming from set *S*.

**Remark 3 .**

Grammatically, both of the intensity and negative words embellish the sentiment words, so the sentiment word is usually in the last position of each element *e*.

### 3.2. Embedding Layer

Compared with the context-independent static word embedding, BERT can generate context-aware dynamic word embedding representation. Thus, we use BERT to obtain word embedding representation for the context and sentimental tendency information. Here, *w* ∈ *ℝ*^*d*^ denotes a real-value word vector, where *d* is the dimension of word embedding. Suppose that the context consists of *n* words, and its corresponding word embedding matrix is denoted as [*w*_1_^*c*^, *w*_2_^*c*^,…, *w*_*n*_^*c*^], where the superscript *c* refers to the term context. Similarly, if the sentimental tendency information has *m* words, its corresponding word embedding matrix is denoted as [*w*_1_^*s*^, *w*_2_^*s*^,…, *w*_*m*_^*s*^]. As shown in Figure 1, these two matrices are the inputs in the IDAN architecture.

### 3.3. Bidirectional LSTM Layer

Because the words in a sentence have strong dependence with their context, we use BiLSTM [36] in this layer. The BiLSTM includes a forward LSTM that reads from the head to end of the sentence and a backward LSTM that reads from the opposite direction. Compared with LSTM, the BiLSTM can get more abundant information. Therefore, we utilize two BiLSTM networks to learn hidden states of context and sentimental tendency information, respectively.

An LSTM cell contains an input gate *i*, a forget gate *f*, an output gate *o*, and a memory cell *c*. In general, at each time step *t*, given the input word embedding *w*_*t*_, previous cell state *c*_*t*−1_, and hidden state *h*_*t*−1_, the current cell state *c*_*t*_ and hidden state *h*_*t*_ in the LSTM networks are updated as

(2) $$\begin{array}{c} X=\left[h_{t-1};w_{t}\right],\\ f_{t}=\sigma \left(W_{f}\cdot X+b_{f}\right),\\ i_{t}=\sigma \left(W_{i}\cdot X+b_{i}\right),\\ o_{t}=\sigma \left(W_{o}\cdot X+b_{o}\right),\\ c_{t}=f_{t}⊙c_{t-1}+i_{t}⊙\;\tan h\left(W_{c}\cdot X+b_{c}\right),\\ h_{t}=o_{t}⊙\;\tan h\left(c_{t}\right), \end{array}$$

where *W*_*f*_, *W*_*i*_, and *W*_*o*_ represent the weight matrix and *b*_*f*_, *b*_*i*_, and *b*_*o*_ represent the bias value learned by the LSTM during the training process. *σ* represents the sigmoid activation function. The symbol · represents matrix multiplication and ⊙ represents element-wise multiplication.

The forward LSTM hidden state ${\overset{⟶}{h}}_{t}$ and backward LSTM hidden state ${\overset{\leftarrow }{h}}_{t}$ at time step *t* in the context part of the model are expressed as

(3) $$\begin{array}{c} {\overset{⟶}{h}}_{t}=\overset{⟶}{\text{LSTM}}\left(w_{t}\right),\;t\in \left[1,n\right], \end{array}$$

(4) $$\begin{array}{c} {\overset{\leftarrow }{h}}_{t}=\overset{\leftarrow }{\text{LSTM}}\left(w_{t}\right),\;t\in \left[n,1\right]. \end{array}$$

Then, the hidden state of BiLSTM at time step *t* is expressed as

(5) $$\begin{array}{c} b_{t}=\left[{\overset{⟶}{h}}_{t}⊕{\overset{\leftarrow }{h}}_{t}\right], \end{array}$$

where the operator ⊕ represents concatenation. After the above operation, we can obtain the contextual semantics representation [*b*_1_^*c*^, *b*_2_^*c*^,…, *b*_*n*_^*c*^] and the sentimental tendency information representation [*b*_1_^*s*^, *b*_2_^*s*^,…, *b*_*m*_^*s*^].

### 3.4. Interaction Layer

After the BiLSTM step, the contextual semantics representation [*b*_1_^*c*^, *b*_2_^*c*^,…, *b*_*n*_^*c*^] and the sentimental tendency information representation [*b*_1_^*s*^, *b*_2_^*s*^,…, *b*_*m*_^*s*^] are obtained. We further use the multihead attention mechanism to learn the interactive representation between the contextual semantics and the sentimental tendency information.

**Figure 2:**
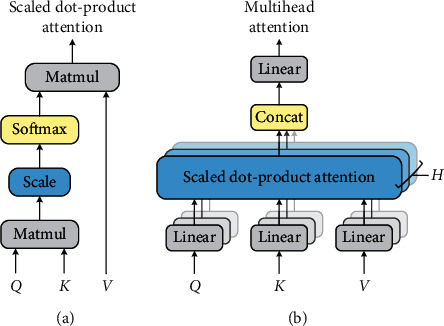
The structure of scaled dot-product attention (a) and multihead attention (b).

The multihead attention is calculated and spliced by multiple scaled dot-product attention, which has three input matrices: Query (*Q*), Key (*K*), and Value (*V*). In the field of NLP, the Key and Value are usually equal [22]; that is, *K*=*V*. The scaled dot-product attention structure is shown in (a) in Figure 2 and is calculated as follows:

(6) $$\begin{array}{c} \text{Attention}\left(Q,K,V\right)=\text{softmax}\left(\frac{QK^{T}}{\sqrt{d_{k}}}\right)V, \end{array}$$

where $1/\sqrt{d_{k}}$ is the scaling factor.  Figure 2(b) shows the structure of multihead attention, which consists of *H* parallel scaled dot-product attention layers. The multihead attention (here denoted by MHA) can be obtained by the following equations:

(7) $$\begin{array}{c} hd_{i}=\text{Attention}\left(QW_{i}^{Q},KW_{i}^{K},VW_{i}^{V}\right), \end{array}$$

(8) $$\begin{array}{c} \text{MHA}\left(Q,K,V\right)=\text{Concat}\left(hd_{1},\ldots ,hd_{H}\right)W^{O}, \end{array}$$

where *W*_*i*_^*Q*^ ∈ *ℝ*^*d*_model_×*d*_*k*_^, *W*_*i*_^*K*^ ∈ *ℝ*^*d*_model_×*d*_*k*_^, *W*_*i*_^*V*^ ∈ *ℝ*^*d*_model_×*d*_*v*_^, and *W*^*O*^ ∈ *ℝ*^*Hd*_*v*_×*d*_model_^ are weight matrices and *d*_model_ denotes the dimension of word hidden representation after BiLSTM processing. *d*_*k*_ and *d*_*v*_ are equal, which denote the dimensions of an attention head. For example, suppose that *d*_model_=1024 and *H*=16; thus, for each attention head, *d*_*k*_=*d*_*v*_=*d*_model_/*H*=64.

**Figure 3:**
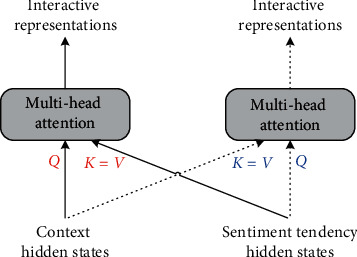
The structure of interactive learning.

In the interactive representation calculation of the context part, the multihead attention has three inputs denoted by *Q*, *K*, and *V*, where *Q* denotes the contextual semantics and *K* and *V* denote the sentimental tendency information.  Figure 3 is the schematic diagram of interactive learning, where the dashed line refers to the calculation process of the interactive representation for sentimental tendency information. Then, we can obtain the interactive representations [*h*_1_^*c*^, *h*_2_^*c*^,…, *h*_*n*_^*c*^] of the contextual semantics and the interactive representations [*h*_1_^*s*^, *h*_2_^*s*^,…, *h*_*m*_^*s*^] of the sentimental tendency information.

### 3.5. Global Attention Layer

In this layer, we use the global attention mechanism to capture the important information of the input sequence and generate an attention representation. As shown in Figure 1, in the context part, the attention infers a variable-length alignment weight vector *α*_*n*_ based on the last output *b*_*n*_^*c*^ of BiLSTM and all output states [*h*_1_^*c*^, *h*_2_^*c*^,…, *h*_*n*_^*c*^] of multihead attention (here denoted by ${\bar{h}}^{c}$). The alignment weight vector *α*_*n*_ is calculated as follows:

(9) $$\begin{array}{c} \alpha_{n}=\frac{\exp\left(\gamma \left(b_{n}^{c},{\bar{h}}^{c}\right)\right)}{\sum_{c^{\prime }}\exp\left(\gamma \left(b_{n}^{c},{\bar{h}}^{c^{\prime }}\right)\right)}, \end{array}$$

where *γ* is a score function that calculates the importance of *h*_*i*_^*c*^ in [*h*_1_^*c*^, *h*_2_^*c*^,…, *h*_*n*_^*c*^]. The score function *γ* is defined as

(10) $$\begin{array}{c} \gamma \left(b_{n}^{c},{\bar{h}}^{c}\right)=b_{n}^{cT}W_{a}{\bar{h}}^{c}, \end{array}$$

where *W*_*a*_ is a weight matrix and *b*_*n*_^*cT*^ is the transpose of *b*_*n*_^*c*^. A global context vector *c*_*n*_ is then computed as follows:

(11) $$\begin{array}{c} c_{n}=\sum_{c}\alpha_{n}{\bar{h}}^{c}. \end{array}$$

Finally, the attention representation *a*_*c*_ in the context part of the model is calculated as follows:

(12) $$\begin{array}{c} a_{c}=f\left(c_{n},b_{n}^{c}\right)=\tan h\left(W_{c}\left[c_{n};b_{n}^{c}\right]\right), \end{array}$$

where tan*h* is a nonlinear activation function and *W*_*c*_ is a weight matrix. Similarly, we can obtain the attention representation *a*_*s*_ of sentimental tendency information.

### 3.6. Output Layer

After attention representations of contextual semantics and sentimental tendency information are obtained, we connect these two vectors into a vector *v* and use it as the input of a linear layer, in which a softmax classifier is implemented for *C* sentiment polarity categories.

The probability with sentiment polarity *i*(*i* ∈ [1, *C*]) is calculated by equations 13) and (14), setting the prediction label to the category with the highest probability value.

(13) $$\begin{array}{c} x=W_{v}v+b_{v}, \end{array}$$

(14) $$\begin{array}{c} y_{i}=\frac{\exp\left(x_{i}\right)}{\sum_{i=1}^{C}\exp\left(x_{i}\right)}, \end{array}$$

where *W*_*v*_ and *b*_*v*_ are weight matrix and bias, respectively, and *y*_*i*_ represents the probability that the input sample belongs to category *i*.

In the model, we denote all network parameters by Φ. Since *L*_2_ regularization can prevent the model from overfitting, we use cross entropy with *L*_2_ regularization as the loss function and try to optimize Φ. The cross-entropy loss function with *L*_2_ regularization is defined as

(15) $$\begin{array}{c} ℒ=-\sum_{t\in T}\sum_{i=1}^{C}g_{i}^{t}\log\left(y_{i}^{t}\right)+\lambda \frac{{\left\|\Phi \right\|}^{2}}{2}, \end{array}$$

where *T* is the training set, *C* is the number of categories, and *g*^*t*^ is the category vector of sample *t*, which is denoted by the one-hot form. *y*_*i*_^*t*^ denotes the distribution of predicted sentiment categories, and *λ* is the regularization coefficient.

In summary, our IDAN neural network shown in Figure 1 can be expressed in a series of equations. Concretely, given the context con and sentimental tendency information sen (obtained by Algorithm 1), the embedding matrices *w*^*c*^ and *w*^*s*^ can be obtained as follows:

(16) $$\begin{array}{c} w^{c},w^{s}=\text{Embedding}\left(\text{con,sen}\right), \end{array}$$

where Embedding represents the embedding layer transformation. Next, the hidden states *b*^*c*^ and *b*^*s*^ can be calculated as follows:

(17) $$\begin{array}{c} b^{c},b^{s}=\text{BiLSTM}\left(w^{c},w^{s}\right), \end{array}$$

where BiLSTM is the bidirectional LSTM layer transformation (implemented by equations (3)–(5)). Then we can get the interactive representations *h*^*c*^ and *h*^*s*^ of the hidden state with respect to the context and sentimental tendency information using the following equation:

(18) $$\begin{array}{c} h^{c},h^{s}=\text{Interaction}\left(b^{c},b^{s}\right), \end{array}$$

where Interaction represents the interaction layer transformation (implemented by equations (7) and (8)). The global attention representations *a*_*c*_ and *a*_*s*_ can be obtained as follows:

(19) $$\begin{array}{c} a_{c},a_{s}=\text{Global}\left(h^{c},h^{s}\right) \end{array}$$

where Global is the global attention layer transformation (implemented by equation (12)). Finally, the sentiment polarity *y*_*i*_ can be calculated as follows:

(20) $$\begin{array}{c} y_{i}=\text{Output}\left(\left[a_{c};a_{s}\right]\right), \end{array}$$

where Output represents the output layer transformation (implemented by equation (13) and (14)).

## 4. Experiments

In this section, four benchmark datasets will be introduced, and then the detail of linguistic resources used in this experiment, evaluation metrics, and hyperparameters setting are given. Next, eight comparable baseline methods will be listed and explained briefly. Finally, the experimental results and analysis are presented, which include performance comparison, ablation experiment, and case analysis.

### 4.1. Datasets

The experiments were evaluated on two Chinese datasets and two English datasets, which are described as follows:

- ChnSentiCorp (available at https://www.aitechclub.com/data-detail?data_id=29): a Chinese hotel review dataset collected by professor Songbo Tan. In the experiment, we chose a balanced corpus containing 6000 reviews that involve positive/negative reviews, which were randomly divided into 80% training set and 20% test set.
- NLPCC-CN (available at http://tcci.ccf.org.cn/conference/2014/pages/page04_sam.html): a Chinese corpus for Task 2 of the 2014 Conference on Natural Language Processing and Chinese Computing (NLPCC), which includes a divided training and a test set. The classification task is positive/negative review discrimination.
- NLPCC-EN (available at http://tcci.ccf.org.cn/conference/2014/pages/page04_sam.html): an English corpus for Task 2 of the 2014 Conference on NLPCC, which involves positive/negative reviews.
- MR (available at https://www.cs.cornell.edu/people/pabo/movie-review-data): a corpus containing movie reviews collected from the IMDB website [37]. The classification task is positive/negative review discrimination. We randomly divided 80% of them as the training set and the remaining 20% as the test set.

The summary of these datasets is shown in Table 1, where *l* represents the average length of the review corpus, |*V*_train_| is the training set size, and |*V*_test_| represents the test set size.

### 4.2. Linguistic Resources

For English data, we utilized linguistic resources published by HowNet (available at http://www.keenage.com/html/c_index.html) to extract sentimental tendency information. For Chinese data, the linguistic resources used to extract sentimental tendency information came from Jianlin Su (available at https://kexue.fm/archives/3360). These resources are summarized in Table 2. It should be noted that each Chinese word was attached to its corresponding English explanation in the following examples.

### 4.3. Evaluation Metrics

We used Accuracy and Macro − *F*1 as evaluation metrics to evaluate the performance of IDAN. Accuracy is one of the most commonly used evaluation metrics in classification tasks, which is defined as follows:

(21) $$\begin{array}{c} \text{Accuracy}=\frac{T}{T+N}, \end{array}$$

where *T* and *N* represent the numbers of samples that the classifier predicted correctly and predicted incorrectly, respectively.

Compared with Accuracy, the Macro − *F*1 score first calculates the Precision and Recall of each category separately. The average of all Precision and Recall is Precision_macro_ and Recall_macro_, respectively. Then, Precision_macro_ and Recall_macro_ are utilized to calculate the Macro − *F*1 score. The calculation formula is as follows:

(22) $$\begin{array}{c} {\text{Precision}}_{\text{macro}}=\frac{1}{C}\overset{C}{\underset{i=1}{\sum }}\frac{{\text{TP}}_{i}}{{\text{TP}}_{i}+{\text{FP}}_{i}},\\ {\text{Recall}}_{\text{macro}}=\frac{1}{C}\overset{C}{\underset{i=1}{\sum }}\frac{{\text{TP}}_{i}}{{\text{TP}}_{i}+{\text{FN}}_{i}},\\ \text{Macro}-F1=\frac{{2\times Precision}_{\text{macro}}{\times Recall}_{\text{macro}}}{{\text{Precision}}_{\text{macro}}{+\text{Recall}}_{\text{macro}}}\text{,} \end{array}$$

where *C* is the number of categories. TP_*i*_, FP_*i*_, and FN_*i*_ are the numbers of true positive, true negative, and false negative of category *i*, respectively.

### 4.4. Hyperparameters Setting

In our experiment, the word embedding of the IDAN model was extracted from the BERT (the English and Chinese pretrained BERT models can be obtained from https://github.com/google-research/bert and https://github.com/ymcui/Chinese-BERT-wwm, respectively) pretraining model with a dimension of 768. The number of neurons in the BiLSTM layer was set to 256, and the number of attention heads of the multihead attention was set to 8. All weight matrices were initialized by Glorot uniform, and all biases were initialized to zero. During the training process, we used the Adam [38] optimization algorithm to train the models with a learning rate of 10^−4^. The batch size was set to 64. To avoid overfitting, a dropout layer with a dropout rate of 0.1 was used before the output layer. The coefficient of *L*_2_ regularization was set to 10^−5^. Besides, we repeated each experiment 10 times and report the average results.

### 4.5. Baseline Methods

We compare IDAN with several baseline methods that are described as follows:

- SVM: a commonly used method in traditional machine learning. In this experiment, the input feature was the average value of the word embeddings of the text sequence.
- LSTM: a layer of LSTM network is used to model the input sequence. Here, we used LSTM's final representation as the input of softmax function for classification.
- BiLSTM: a layer of BiLSTM network is used to model the input sequence. We used BiLSTM's final representation as the input of softmax function for classification instead of pooling after obtaining the hidden state of each word.
- ATT-BiLSTM: the attention mechanism is attached on the basis of a layer of BiLSTM network. After using BiLSTM to obtain the hidden state of each word, these hidden state representations were the input of the attention module.
- H-RNN-CNN [9]: a multilayer network structure for processing Chinese text sentiment classification tasks, in which the input text was divided into sentences as the input of a middle layer to address the problem of information loss that may be caused by long text. In the model, LSTM was utilized to process context sequences, and CNN was used to capture the relationship among sentences.
- CRNN [29]: an architecture combining CNN and RNN (LSTM and GRU), which takes advantage of the coarse-grained local features generated by CNN and long-distance dependencies learned via RNN for short texts.
- fastText [39]: a simple and efficient text classification method. It utilizes the average word vector of the n-gram features of the text and hierarchical softmax for classification.
- LR-BiLSTM [16]: a linguistically regularized BiLSTM model, which integrates sentiment, negative, and intensity words into BiLSTM to address the sentiment shifting effect of these words.
- Furthermore, we have designed several ablation experiments to illustrate the effectiveness of IDAN.
- IDAN-W2V: use pretrained Word2Vec (the English and Chinese pretrained Word2Vec models can obtained from https://code.google.com/archive/p/word2vec/ and https://github.com/Embedding/Chinese-Word-Vectors, respectively) [40] as word embedding instead of extracting from the BERT. The purpose of this experiment is to demonstrate the advantage of BERT in IDAN.
- IDAN-NSTI: only the original text was used for sentiment classification without considering the sentimental tendency information (i.e., only use the context part in IDAN).
- IDAN-NIL: there is no interactive learning in IDAN, which means that contextual semantics and sentimental tendency information are not related to each other before the final concatenation.
- IDAN-NGA: there is no global attention in IDAN, and the output of multihead attention is used as the input of softmax after average pooling operation.

### 4.6. Results and Analysis

Here, we first give the performance comparison with the baseline methods described above. Then, we conduct the ablation study experiment, which aims to explore why the network architecture of IDAN can work well, where the symbol “-” denotes being not reported, and the best performers are in bold. Finally, two visualization cases are presented to illustrate the relationship between attention weight distribution and sentiment polarity of words.

#### 4.6.1. Performance Comparison with Baseline Models

The performance comparison results are given in Table 3, where SVM performs the worst on the ChnSentiCorp, NLPCC-CN, and NLPCC-EN datasets but is better than LSTM on the MR dataset. This may be related to the situation where sequences on the first three datasets are longer and more complex for SVM. Compared with LSTM, the accuracy of BiLSTM on the ChnSentiCorp, NLPCC-CN, NLPCC-EN, and MR datasets is improved by 1.5%, 0.31%, 1.07%, and 0.97%, respectively. The possible reason is that BiLSTM can capture contextual information from two directions. Since the attention mechanism can assign different attention weight to each word, it can be seen that the performance of ATT-BiLSTM is improved a little bit compared with BiLSTM on all datasets. Besides, although H-RNN-CNN uses two layers of LSTM to model sentences and uses CNN to capture cross-sentence information, its accuracy is higher than ATT-BiLSTM on the MR dataset but is lower on the ChnSentiCorp and NLPCC-CN datasets. Compared with H-RNN-CNN, the performance of CRNN is improved by about 1%. This is because CRNN not only uses multiple CNNs of different sizes to extract the local features of the sequence but also uses LSTM or GRU to capture the long-term dependence of the sequence.

As a simple method, fastText achieves a comparable result with CRNN. Its accuracies on the ChnSentiCorp and NLPCC-EN datasets are even higher than CRNN by about 0.95% and 0.91%, respectively. Although the LR-BiLSTM model incorporates linguistic resources and obtains good performance on the MR dataset, its accuracy is higher than that of fastText by about 0.29% but lower than CRNN by about 0.18%. This may be due to the fact that LR-BiLSTM did not make full use of linguistic resources.

As can be seen, our IDAN model performs best on all datasets. Compared with the best baseline model, the accuracies of IDAN on the ChnSentiCorp, NLPCC-CN, NLPCC-EN, and MR datasets are improved by 0.94%, 2.99%, 5.11%, and 0.38%, respectively, demonstrating the effectiveness of our proposed method.

#### 4.6.2. Ablation Experiments

The ablation experiment result is shown in Table 4. Firstly, we compared the experimental performance while using different pretrained word vectors. In IDAN-W2V, BERT embedding was replaced by Word2Vec, which results in a significant decrease in performance compared to IDAN. However, it is noteworthy that the performance of IDAN-W2V is still comparable to CRNN. Similarly, when interactive learning between contextual semantics and sentimental tendency information is not implemented, the experimental performance is slightly degraded on all the datasets. Secondly, when we separately ablate the sentimental tendency information part and global attention layer of the full model, its performance will degrade on the ChnSentiCorp, NLPCC-EN, and MR datasets. Particularly, when the global attention layer is ablated, the best result of ablation experiments on the NLPCC-CN dataset can be achieved.

These ablation experiments show that the performance of IDAN-W2V is comparable to the baseline model CRNN. However, it has a relatively large gap compared with the performance of the full model. Overall, the situations of IDAN-NIL, IDAN-NSTI, and IDAN-NGA are relatively similar, and their performance is better than IDAN-W2V but slightly lower than the full model.

These results indicate that the BERT embedding has brought about a considerable performance improvement to our method. Moreover, extracting the sentimental tendency information, learning the interactive representation, and the global attention layer also help improve the classification performance.

#### 4.6.3. Case Analysis

**Figure 4:**
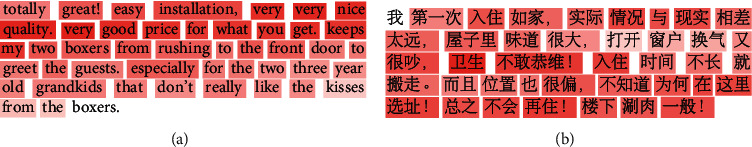
Visualization of attention weights for two test cases: (a) case 1 and (b) case 2.

In this section, an English review text on the NLPCC-EN dataset and a Chinese review text on the ChnSentiCorp dataset are used as the case analysis.  Figure 4 is the visualization result of the attention weights calculated by equation (9) for two test cases. Here, the color concentration reflects the attention weight of the corresponding word, that is, the importance of words. The sentiment polarity of Figure 4(a) is positive, and that of Figure 4(b) is negative. Both Figures 4(a) and 4(b) are predicted correctly by the IDAN model.

From the weight distribution of attention in Figure 4(a), it can be seen that the model assigns greater weight to words or phrases with strong positive sentiment, such as *“very very nice quality”* and *“very good price for what you get*.*”* In Figure 4(b), the model assigns greater weight to words and phrases with strong negative sentiment, such as* (meaning: poor sanitary conditions)”*and* (meaning: will not stay at the hotel again)”*. This attention weight distribution illustrates that our model can effectively focus on words or phrases that are important for sentiment polarity.

## 5. Conclusion and Future Work

In this paper, we propose a novel model called Interactive Dual Attention Network (IDAN), which can utilize the interaction between contextual semantics and sentimental tendency information for sentiment classification. We design an algorithm to obtain sentimental tendency information and extract the BERT embedding as the model embedding layer. We also use BiLSTM networks to learn the dependencies of contextual semantics and sentimental tendency information, respectively. Finally, multihead attention is used to implement interaction, and global attention is utilized to focus on the important parts of the sequence and to generate the final representation for the classifier. Extensive experiments conducted on four benchmark datasets show that our method is effective and outperforms the competition baseline methods. Furthermore, ablation experiments illustrate that BERT embedding has brought about a considerable performance improvement. Meanwhile, extracting the sentimental tendency information for the interactive representation also contributes to performance improvement.

For future work, improving the algorithm for extracting sentimental tendency information and optimizing the interactive attention network may further improve the classification performance and obtain more interpretability. Furthermore, we also plan to introduce more refining linguistic knowledge into the network to make the model be more discriminative and robust.

## Figures and Tables

**Algorithm 1:**
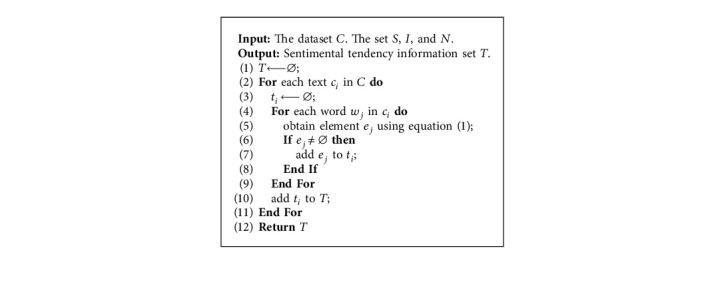
Sentimental tendency information extraction.

**Table 1:** Summary of the datasets after tokenization.

Dataset	*l*	|*V*_train_|		|*V*_test_|	
		Positive	Negative	Positive	Negative
ChnSentiCorp	136	2400	2400	600	600
NLPCC-CN	64	5000	5000	1250	1250
NLPCC-EN	130	4987	4998	1250	1250
MR	20	4264	4264	1067	1067

**Table 2:** Summary of the lexicons used in the experiments.

Language	Lexicon types	Words count	Examples
English	Positive sentiment	4363	Applause, satisfied
	Negative sentiment	4572	Abuse, get sick of
	Intensity words	171	Absolutely, ultra

Chinese	Positive sentiment	10191	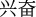 (excitement), 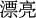 (beautiful)
	Negative sentiment	13712	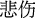 (sadness), 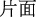 (one-sided)
	Intensity words	79	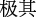 (extremely), 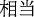 (fairly)
	Negative words	71	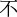 (no), 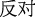 (against)

**Table 3:** Performance comparison with baseline methods.

Approach	ChnSentiCorp		NLPCC-CN		NLPCC-EN		MR	
	Accuracy	Macro-*F*1	Accuracy	Macro-*F*1	Accuracy	Macro-*F*1	Accuracy	Macro-*F*1
SVM	0.8618	0.8528	0.7479	0.7441	0.8226	0.8143	0.7914	0.7852
LSTM	0.8681	0.8570	0.7572	0.7557	0.8381	0.8379	0.7844	0.7705
BiLSTM	0.8831	0.8693	0.7603	0.7573	0.8488	0.8477	0.7941	0.7877
ATT-BiLSTM	0.8945	0.8892	0.7665	0.7585	0.8503	0.8491	0.7952	0.7909
H-RNN-CNN	0.8940	0.9030	0.7550	0.7790	—	—	0.8190	—
CRNN	0.9108	0.9082	0.7702	0.7648	0.8579	0.8456	0.8228	—
fastText	0.9203	0.9170	0.7706	0.7624	0.8670	0.8615	0.8181	0.8121
LR-BiLSTM	—	—	—	—	—	—	0.8210	—
IDAN	**0.9297**	**0.9293**	**0.8005**	**0.7875**	**0.9181**	**0.9068**	**0.8266**	**0.8135**

Bold values indicate the best performances.

**Table 4:** Results for the ablation experiments.

Approach	ChnSentiCorp		NLPCC-CN		NLPCC-EN		MR	
	Accuracy	Macro-*F*1	Accuracy	Macro-*F*1	Accuracy	Macro-*F*1	Accuracy	Macro-*F*1
IDAN-W2V	0.9145	0.9078	0.7667	0.7657	0.8621	0.8515	0.7986	0.7870
IDAN-NIL	0.9262	0.9141	0.8002	0.7866	0.9155	0.9069	0.8214	0.8100
IDAN-NSTI	0.9233	0.9099	0.8045	0.7911	0.9128	0.9062	0.8225	0.8134
IDAN-NGA	0.9184	0.9133	**0.8067**	**0.7920**	0.9164	0.9068	0.8254	0.8130
IDAN	**0.9297**	**0.9293**	0.8005	0.7875	**0.9181**	**0.9145**	**0.8266**	**0.8135**

Bold values indicate the best performances.

## Data Availability

The data and the authors' source code used to support the findings of this study will be available at https://github.com/zhuyl96/IDAN.
